# Identifying mixed *Mycobacterium tuberculosis* infections from whole genome sequence data

**DOI:** 10.1186/s12864-018-4988-z

**Published:** 2018-08-14

**Authors:** Benjamin Sobkowiak, Judith R. Glynn, Rein M. G. J. Houben, Kim Mallard, Jody E. Phelan, José Afonso Guerra-Assunção, Louis Banda, Themba Mzembe, Miguel Viveiros, Ruth McNerney, Julian Parkhill, Amelia C. Crampin, Taane G. Clark

**Affiliations:** 1 Faculty of Infectious and Tropical Diseases, London School of Hygiene & Tropical Medicine, London, UK; 2 Faculty of Epidemiology and Population Health, London School of Hygiene & Tropical Medicine, London, UK; 3 TB Modelling Group, TB Centre, London School of Hygiene and Tropical Medicine, London, UK; 4 Bill Lyons Informatics Centre, University College London, London, UK; 5 Karonga Prevention Study, Chilumba, Malawi; 6 Global Health and Tropical Medicine, Instituto de Higiene e Medicina Tropical, Universidade Nova de Lisboa, Lisbon, Portugal; 7 Wellcome Trust Sanger Institute, Hinxton, UK

**Keywords:** Mycobacterium tuberculosis, Tuberculosis, Bioinformatics, Epidemiology, Genomic analysis, Mixed infection

## Abstract

**Background:**

Mixed, polyclonal *Mycobacterium tuberculosis* infection occurs in natural populations. Developing an effective method for detecting such cases is important in measuring the success of treatment and reconstruction of transmission between patients. Using whole genome sequence (WGS) data, we assess two methods for detecting mixed infection: (i) a combination of the number of heterozygous sites and the proportion of heterozygous sites to total SNPs, and (ii) Bayesian model-based clustering of allele frequencies from sequencing reads at heterozygous sites.

**Results:**

*In silico* and *in vitro* artificially mixed and known pure *M. tuberculosis* samples were analysed to determine the specificity and sensitivity of each method. We found that both approaches were effective in distinguishing between pure strains and mixed infection where there was relatively high (> 10%) proportion of a minor strain in the mixture. A large dataset of clinical isolates (*n* = 1963) from the Karonga Prevention Study in Northern Malawi was tested to examine correlations with patient characteristics and outcomes with mixed infection. The frequency of mixed infection in the population was found to be around 10%, with an association with year of diagnosis, but no association with age, sex, HIV status or previous tuberculosis.

**Conclusions:**

Mixed *Mycobacterium tuberculosis* infection was identified in silico using whole genome sequence data. The methods presented here can be applied to population-wide analyses of tuberculosis to estimate the frequency of mixed infection, and to identify individual cases of mixed infections. These cases are important when considering the evolution and transmission of the disease, and in patient treatment.

**Electronic supplementary material:**

The online version of this article (10.1186/s12864-018-4988-z) contains supplementary material, which is available to authorized users.

## Background

The innovation of whole genome sequencing (WGS) has brought about significant developments in our understanding of bacterial disease dynamics, including the population-level transmission of pathogens and the spread of antimicrobial resistance [1–3]. Typically, studies consider a single consensus genome to be representative of an infection. Often variation between pathogens is determined by the comparison of genetic variants, such as single nucleotide polymorphisms (SNPs). However, analysis of these variants can identify more than one allele present at a single locus, resulting in a heterozygous base call in haploid bacterial genomes. These sites are usually excluded from further analysis: they can represent sequencing errors, but heterozygous calls may be biologically relevant and indicate the presence of mixed infection [4–6].

Mixed infection occurs when two or more strains of the same species of pathogen are present in an individual host at any one time. Strain heterogeneity arises from transmission from multiple sources to a recipient and is distinct from clonal evolution within the host [4]. A failure to identify the entire within-host pathogen diversity can impact treatment and clinical outcomes, with undetected strains potentially possessing key phenotypic differences such as antibiotic resistance and virulence [4], or being misinterpreted as reinfections rather than relapses. Additionally, attempts to reconstruct the transmission of bacterial pathogens can be complicated as only one strain of a mixed infection may be represented and true transmission links may not be established [5].

Polyclonal, mixed *M. tuberculosis* infections occur in natural populations and have been linked to high incidence populations with an elevated chance of exposure [6–9]. Previous attempts to determine the presence of mixed *M. tuberculosis* infections have primarily focused on polymerase chain reaction (PCR) based techniques such as *IS6110* restriction fragment length polymorphism (RFLP) and MIRU-VNTR to look for heterogeneity at diagnostic loci [7–11]. These approaches, though, can only detect strains that are relatively distant genetically and require a high proportion of minor variants in the sample [12].

Strain heterogeneity has also been studied between single colonies grown from single sputum samples [11, 13]. Culturing can reduce the number of strains identified through differential survival through serial rounds of culture and subsequent growth on solid media [6, 14]. Additionally, taking single samples from one site will not account for potential strain heterogeneity across different sites, which has been revealed through sequencing strains from multiple biopsies in the lung [15]. Phylogenetic approaches revealing multiple divergent paths of heterogeneous SNPs have been more successful at detecting mixed *M. tuberculosis* infections [16], though this method can be limited by the robustness of evolutionary tree and ancestral state reconstruction. A maximum-likelihood approach has been employed in one study using the allele frequency at mixed sites in whole genome sequences of *Clostridium difficile* [4]. This method was effective at identifying two-strain mixed infections determined using a previously characterised database of haplotypes.

We aim to develop a simple method for detecting non-clonal mixed infections of *M. tuberculosis* and estimate mixture proportions from whole genome sequence data alone. We use a test dataset of 48 *in vitro* and 168 in silico artificial mixtures in known proportions to develop an approach for identifying mixed samples and determining mixture proportions from whole genome sequencing data. These methods are refined and tested further using replicate tuberculosis (TB) samples from Portugal and five replicate H37Rv reference strain samples. Finally, we apply the resulting methods to an extensive clinical set of 1963 *M. tuberculosis* strains isolated from patients in Malawi, a high-burden TB + HIV setting [17], with a high TB incidence [18, 19]. In this setting we assess the prevalence of mixed infection in an unselected population, and examine correlations with patient characteristics and outcomes.

## Methods

### Sample preparation and sequencing

Over 2000 *Mycobacterium tuberculosis* samples were obtained from TB patients recruited as part of the Karonga Prevention Study in northern Malawi, which has been conducting research on mycobacterial infections in the region since the 1980s. Patients exhibiting symptoms of TB are reviewed by project staff at the district hospital and local health centres, with those diagnosed with the disease interviewed to obtain further patient details. Information collected includes sex, age, HIV status and contact with prior cases. A minimum of three sputum samples were taken from each patient. The studies were approved by the Health Sciences Research Committee in Malawi and by the London School of Hygiene and Tropical medicine ethics committee. HIV testing included pre- and post-test counselling and informed consent. Written consent was sought and obtained for all studies. Whole genome sequencing was carried out on DNA extracted using extraction kits from a sweep of multiple colonies from solid cultures for all Malawi samples using the Illumina HiSeq 2000 platform generating 100 base-pair paired-end reads. After sequencing and quality control, 1963 whole genome sequences were available for analysis.

Forty-eight mixed *M. tuberculosis* samples were artificially generated *in vitro* by combining DNA from two clinical cultures of *M. tuberculosis* from the Malawi patients. The DNA is quantified through spectrophotometry in liquid culture and mixed in the appropriate volume to produce mixed samples with the majority/minority strain proportions 0.70/0.30, 0.90/0.10, 0.95/0.05, and 1.00/0.00, before sequencing on the Illumina HiSeq 2000 platform (Table 1). The paired strains encompassed both between- and within-lineage mixes covering the four major ancient and contemporary lineages, 1–4, in *M. tuberculosis,* including Beijing strain-types (lineage 2).

**Table 1:** Detection of artificially mixed infections using the number of heterozygous SNPs and Bayesian model-based clustering analysis methods. Strain information, known mixture proportions and average coverage across the genome are also shown. The number of heterozygous SNPs in each sample is presented with the total number of different distinct coding and non-coding regions in which the SNPs are present

	Sample information				Heterozygous sites to total SNP proportion			Bayesian model-based clustering	
Sample identifier	Major strain proportion	Major/minor strain lineage	Major/minor strain spoligotype	Average coverage	No. heterozygous SNPs (Total no. gene regions)	No. total SNPs	Proportion het-total SNPs (%)	No. of strains	Major strain proportion
ERR221663	**0.7**	**1/3**	**EAI6-BGD1/CAS1-Kili**	**183**	**1789 (1382)**	**2372**	**75.4**	**2**	**0.64**
ERR221662	**0.7**	**3/1**	**CAS1-Kili/EAI6-BGD1**	**176**	**1788 (1381)**	**2373**	**75.3**	**2**	**0.72**
ERR221641	**0.7**	**4/3**	**CAS1-Delhi/LAM11-ZWE**	**179**	**1055 (912)**	**1443**	**73.1**	**2**	**0.68**
ERR221643	**0.7**	**3/4**	**LAM11-ZWE/CAS1-Delhi**	**157**	**1051 (912)**	**1437**	**73.1**	**2**	**0.69**
ERR221656	**0.7**	**2/4**	**Beijing/LAM11-ZWE**	**179**	**1046 (906)**	**1426**	**73.4**	**2**	**0.69**
ERR221659	**0.7**	**4/2**	**LAM11-ZWE/Beijing**	**196**	**1042 (906)**	**1415**	**73.6**	**2**	**0.71**
ERR221623	**0.7**	**1/1**	**EAI1-SOM/EAI6-BGD1**	**172**	**988 (848)**	**2271**	**43.5**	**2**	**0.73**
ERR221627	**0.7**	**1/1**	**EAI6-BGD1/EAI1-SOM**	**189**	**985 (845)**	**2269**	**43.4**	**2**	**0.65**
ERR221647	**0.7**	**4/4**	**LAM11-ZWE/T**	**223**	**640 (571)**	**784**	**81.6**	**2**	**0.72**
ERR221651	**0.7**	**4/4**	**T/LAM11-ZWE**	**175**	**636 (567)**	**779**	**81.6**	**2**	**0.67**
ERR221630	**0.7**	**2/2**	**Beijing/Beijing**	**168**	**186 (181)**	**1212**	**15.3**	**2**	**0.67**
ERR221628	**0.7**	**2/2**	**Beijing/Beijing**	**191**	**183 (178)**	**1212**	**15.1**	**2**	**0.69**
ERR221660	**0.9**	**1/3**	**EAI6-BGD1/CAS1-Kili**	**211**	**617 (550)**	**1932**	**31.9**	**2**	**0.85**
ERR221620	**0.9**	**1/1**	**EAI6-BGD1/EAI1-SOM**	**173**	**571 (520)**	**1994**	**28.6**	**2**	**0.85**
ERR221636	**0.9**	**3/4**	**LAM11-ZWE/CAS1-Delhi**	**155**	**316 (299)**	**893**	**35.4**	**2**	**0.86**
ERR221665	**0.9**	**3/1**	**CAS1-Kili/EAI6-BGD1**	**151**	**232 (218)**	**1299**	**17.9**	**2**	**0.87**
ERR221640	**0.9**	**4/3**	**CAS1-Delhi/LAM11-ZWE**	**162**	**169 (155)**	**1175**	**14.4**	**2**	**0.87**
ERR221654	**0.9**	**4/2**	**LAM11-ZWE/Beijing**	**205**	**165 (156)**	**772**	**21.4**	**2**	**0.86**
ERR221644	**0.9**	**4/4**	**T/LAM11-ZWE**	**181**	**150 (143)**	**360**	**41.7**	**2**	**0.86**
ERR221625	**0.9**	**1/1**	**EAI1-SOM/EAI6-BGD1**	**168**	**147 (141)**	**1814**	**8.1**	**2**	**0.86**
ERR221652	**0.9**	**2/4**	**Beijing/LAM11-ZWE**	**192**	**138 (130)**	**1139**	**12.1**	**2**	**0.86**
ERR221634	**0.9**	**2/2**	**Beijing/Beijing**	**177**	**72 (69)**	**1137**	**6.3**	**2**	**0.85**
ERR221629	**0.9**	**2/2**	**Beijing/Beijing**	**170**	**55 (55)**	**1130**	**4.9**	**2**	**0.86**
ERR221649	**0.9**	**4/4**	**LAM11-ZWE/T**	**169**	**12 (10)**	**687**	**1.7**	**1**	**1**
ERR221635	**0.95**	**2/2**	**Beijing/Beijing**	**193**	**609 (535)**	**1162**	**52.4**	**2**	**0.83**
ERR221632	**0.95**	**2/2**	**Beijing/Beijing**	**196**	**186 (177)**	**1136**	**16.4**	**2**	**0.85**
ERR221653	**0.95**	**4/2**	**LAM11-ZWE/Beijing**	**170**	**20 (16)**	**699**	**2.9**	**1**	**1.00**
ERR221650	**0.95**	**4/4**	**LAM11-ZWE/T**	**174**	**18 (18)**	**691**	**2.6**	**1**	**1.00**
ERR221637	**0.95**	**4/3**	**CAS1-Delhi/LAM11-ZWE**	**156**	**29 (19)**	**1148**	**2.5**	**1**	**1.00**
ERR221645	0.95	4/4	T/LAM11-ZWE	**200**	6 (4)	241	**2.5**	1	**1.00**
ERR221655	**0.95**	**2/4**	**Beijing/LAM11-ZWE**	**193**	**20 (17)**	**1117**	**1.8**	**2**	**0.87**
ERR221664	**0.95**	**3/1**	**CAS1-Kili/EAI6-BGD1**	**166**	**18 (9)**	**1185**	**1.5**	**1**	**1.00**
ERR221666	**0.95**	**1/3**	**EAI6-BGD1/CAS1-Kili**	**157**	**25 (20)**	**1774**	**1.4**	**2**	**0.91**
ERR221626	**0.95**	**1/1**	**EAI1-SOM/EAI6-BGD1**	**189**	**20 (18)**	**1766**	**1.1**	**1**	**1.00**
ERR221638	0.95	3/4	LAM11-ZWE/CAS1-Delhi	**161**	6 (6)	678	**0.9**	1	**1.00**
ERR221621	0.95	1/1	EAI6-BGD1/EAI1-SOM	**174**	12 (11)	1792	**0.7**	1	**1.00**
ERR221646	1.00	4	T	**165**	4 (2)	242	**1.7**	1	**1.00**
ERR221642	1.00	4	CAS1-Delhi	**170**	12 (5)	1144	**1.1**	1	**1.00**
ERR221624	1.00	1	EAI1-SOM	**187**	18 (7)	1765	**1.0**	1	**1.00**
ERR221648	1.00	4	LAM11-ZWE	**190**	7 (6)	685	**1.0**	1	**1.00**
ERR221657	1.00	4	LAM11-ZWE	**173**	7 (5)	687	**1.0**	1	**1.00**
ERR221661	1.00	3	CAS1-Kili	**178**	11 (7)	1185	**0.9**	1	**1.00**
ERR221633	1.00	2	Beijing	**171**	5 (5)	1110	**0.5**	1	**1.00**
ERR221658	1.00	2	Beijing	**185**	5 (5)	1113	**0.5**	1	**1.00**
ERR221631	1.00	2	Beijing	**151**	5 (5)	1129	**0.4**	1	**1.00**
ERR221639	1.00	3	LAM11-ZWE	**147**	2 (2)	671	**0.3**	1	**1.00**
ERR221667	1.00	1	EAI6-BGD1	**180**	4 (3)	1769	**0.2**	1	**1.00**
ERR221622	1.00	1	EAI6-BGD1	**187**	3 (3)	1789	**0.2**	1	**1.00**

The samples are ordered by the known major strain proportion and then by number of heterozygous sites. Samples identified as mixed infections are shown in bold

Portuguese *M. tuberculosis* clinical isolates were sourced from ten patients with known drug-resistant TB admitted to four different hospitals in Lisbon between 2007 and 2013, with written consent obtained. All clinical strains and the reference strain H37Rv (ATCC 25618D-9, Lot # 60986340) and their replicates were prepared by inoculating a single colony into Middlebrook 7H9 broth supplemented with 10% OADC (Oleic Albumin Dextrose Catalase) (Becton Dickinson). Cultures underwent whole genome sequencing using MiSeq technology (as described in Phelan et al. [20]).

### Variant calling

Sequenced reads were quality checked using *FastQC* and trimmed to remove adapter sequences and low quality reads using *trimmomatic* [21]. Reads were mapped to the H37Rv reference strain (Genbank no.: NC_000962.3) using *BWA-mem* [22]. Variant calling was conducted using *SAMtools* and *BCFtools* [23], with low quality variants (Phred score Q < 20, combined depth DP < 10) removed and heterozygous calls enabled. Additionally, variants were removed from the analysis if there was no call (either through non-alignment of reads or low coverage) in > 10% of individuals.

### In silico simulated mixed infections

A dataset of 168 artificial *M. tuberculosis* mixtures were produced in silico by simulating whole genome sequences in the FASTQ format from consensus sequences of eight Malawi clinically derived samples, two from each lineage 1–4, using *DWGSIM* software [24]. The sequencing error rate was set as 0.0026 for forward reads and 0.0040 for reverse reads reflecting the true error rates of Illumina HiSeq sequencing [25] and the average genome-wide substitution rate set as 1 × 10^− 7^. Sequence files were combined to produce mixed samples with the majority/minority strain proportions 0.70/0.30, 0.90/0.10, 0.95/0.05 of both between- and within-lineage mixes and mean coverage of 100× across the genome (Additional file 1).

### Characterising heterozygous base calls

Heterozygous base calls were considered informative for determining mixed infections. In mixed infection samples, mapped sequences at these sites will be a combination of reads from one strain carrying a SNP at this position and reads from one or more additional strains that do not, resulting in more than one allele call. While the presence of these heterozygous base calls can be indicative of strain mixing, these calls can also be present in the variant output of non-mixed clonal samples at sites under strong selection, or in regions of high variability. SNPs in *pe/ppe* gene regions and known antibiotic resistance determining genes were excluded from the analysis to remove sites that are more likely to result in heterozygous calls in non-mixed populations. Furthermore, to distinguish between clonal heterogeneity and true mixed infections, only samples with > 10 heterozygous sites will be considered potential mixed infections in further analysis. This estimate has been calculated in previous work with the Malawi samples, with up to 10 SNPs present between individuals in chains of transmission or found within individuals evolving over time [26].

### Detecting mixed infection using the heterozygous base calls

The first approach to detect mixed infection used the number of heterozygous base calls across the genome to set a minimum threshold for distinguishing mixtures (denoted as the “heterozygous sites method”). In samples that were close to the determined threshold, we included a measure of the proportion of heterozygous calls to total SNPs to further distinguish between mixed and pure samples. This approach will help to correctly identify pure samples that have a relatively high level of variation across the genome. This simple method allows for rapid identification of potential mixtures in large datasets without requiring the more complex interrogation of the sequence reads to calculate allele frequencies at heterozygous sites. The threshold at which samples were considered mixtures was determined using the *in vitro* mixed samples, the analysis of which was blind to the known mixture proportion of each sample, to determine whether an effective cut-off could be established from variant calling alone.

### Detecting mixed infection with Bayesian model-based clustering

**Fig. 1:**
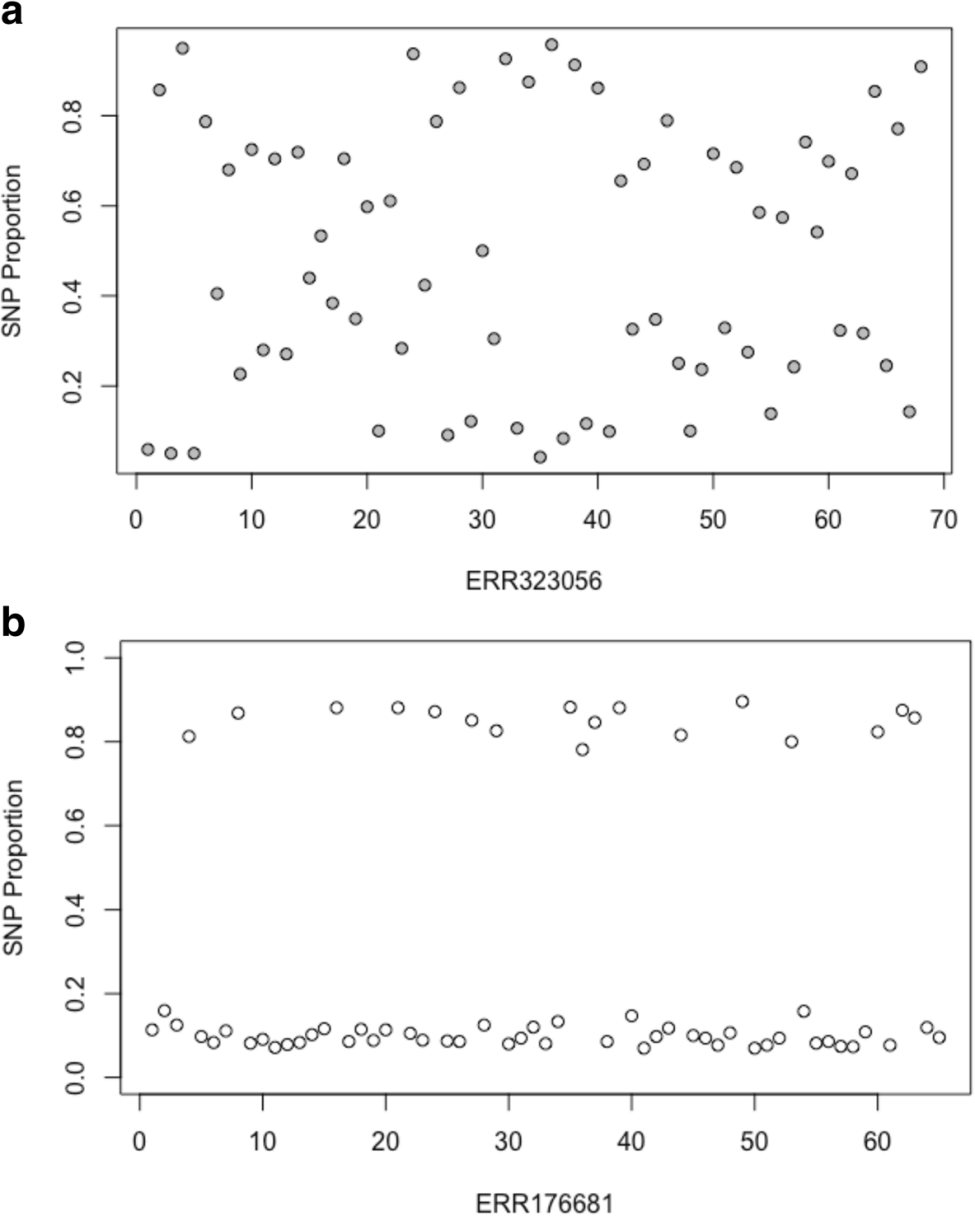
Heterozygous SNP plots for two clinical Malawi samples, illustrating the difference between clonal heterogeneity (**a**) and the signals of mixed infections (**b**). The x-axis represents contiguous SNPs across the genome (numbered sequentially) with heterozygous SNP calls, and the y-axis represents the proportion of non-reference alleles at that SNP. **a** shows no evidence of mixed infection, with read frequencies at heterozygous sites randomly distributed between 0 and 1. **b** demonstrates the characteristic pattern of mixed infection with two different strains, with the read frequencies clustering into two distinct clusters with means around 0.90 and 0.10, implying a 0.9/0.1 mixture

An alternative approach for detecting mixed infection was employed that estimated the number of strains present in a sample through Bayesian model-based clustering of allele frequencies at heterozygous sites, implemented through the *mclust* package in *R* [27]*.* A Bayesian model was employed to minimise the impact of outlier data points that can affect the direction and classification of clustering groups when using other methods such as principal component analysis (PCA) [28]. For each sample, the major and minor allele frequencies of mapped reads at each heterozygous base call was calculated (removing reads where the base call has low sequence quality (Phred P_error > 0.05)) and used as a univariate input for clustering. The allele frequencies of heterozygous sites in mixed infection samples will cluster at similar frequencies in a set number of groups depending on the number and proportion of strains present. On the other hand, the allele frequencies of heterozygous sites in pure samples, though there may be a high number of heterozygous sites in samples with high clonal heterogeneity, will be more randomly distributed without clustering. These differences are illustrated in Fig. 1.

Our model aimed to determine if the allele frequencies of heterozygous sites in a sample can be optimally clustered into groups relating to mixed infections of two strains, or if the sample is a non-mixed, pure strain. Though our methods were developed for identifying mixed samples of two strains, the model can, in theory, be extended to search for higher numbers of strains in a mixture. The *Mclust* function in the *mclust* package in R works to determine the likelihood of the data coming from a distribution with a set number of clusters, or mixture components, specified as *G*. The probability of each observation coming from a mixture component is modelled by a Gaussian distribution, with each group described by the mean and unequal, scalar variance. The likelihood of *G* was assessed through the Bayesian information criterion (BIC) value of model selection. Underlying model calculations are shown elsewhere [27].

The model was applied to all samples to identify the optimal number of clustering groups (*G =* 2 is characteristic of two-strain mixed infections), with the model likelihood assessed through the resulting BIC value. A sample is classified as being a mixed infection of two strains (*G* = 2) where, (i) the number of heterozygous sites is > 10, and (ii) the BIC value of *G* = 2 is > 20. The BIC value threshold for *G* = 2 was obtained from analysing the artificially mixed *in vitro* samples and is explained in more detail in the Results section of this paper. This method could be extended to identify mixed infections of more than two strains where the optimal number of found to be greater than two, though none of our data fulfilled this criterion. Samples were classified as likely containing a single strain (unmixed) where, (i) the number of heterozygous sites is ≤10 or (ii) the number of heterozygous sites is > 10 but the BIC value for *G =* 2 was lower than the threshold.

## Results

### In vitro artificially mixed M. Tuberculosis samples

Table 1 shows the sample information for each artificial mixture along with the results of both mixture detection approaches, arranged by the known major strain proportion and then by the number of heterozygous sites.

For the heterozygous sites method, a clear threshold that discriminates between mixed samples and pure strains was not attained with our analysis, though with a heterozygous SNP threshold of ≥20 sites, all but one samples with a major proportion of 0.70 (12/12) and 0.90 (11/12) were correctly classifies as mixed, and all non-mixed samples as pure (12/12). Introducing an additional condition of > 1.5% heterozygous to total SNP proportion for samples containing between 11 and 19 heterozygous sites correctly identifies the 0.90 major proportion sample with less than 20 heterozygous sites (ERR221649) as a mixed infection, with still no pure samples incorrectly classified.

Mixtures of 0.95/0.05 were more difficult to discriminate from non-mixtures, with only 9/12 mixed samples correctly identified using the combined thresholds of i) ≥ 20 heterozygous sites and ii) > 1.5% heterozygous sites to total SNP proportion in samples with 11–19 heterozygous sites. The number of heterozygous varied considerably within these mixes between 609 and 6 sites. One 0.95/0.05 sample had a heterozygous proportion over 1.5% but contained only 6 heterozygous sites so was indistinguishable from clonal variation. Eleven of the twelve pure strains had a heterozygous proportion under 1.5%, with the other pure sample identified as non-mixed through the low number of heterozygous sites (4 SNPs). Thus, this method correctly identifies 33/36 mixed infections with no false positive results.

### Identifying *in vitro* mixtures through Bayesian model-based clustering

**Fig. 2:**
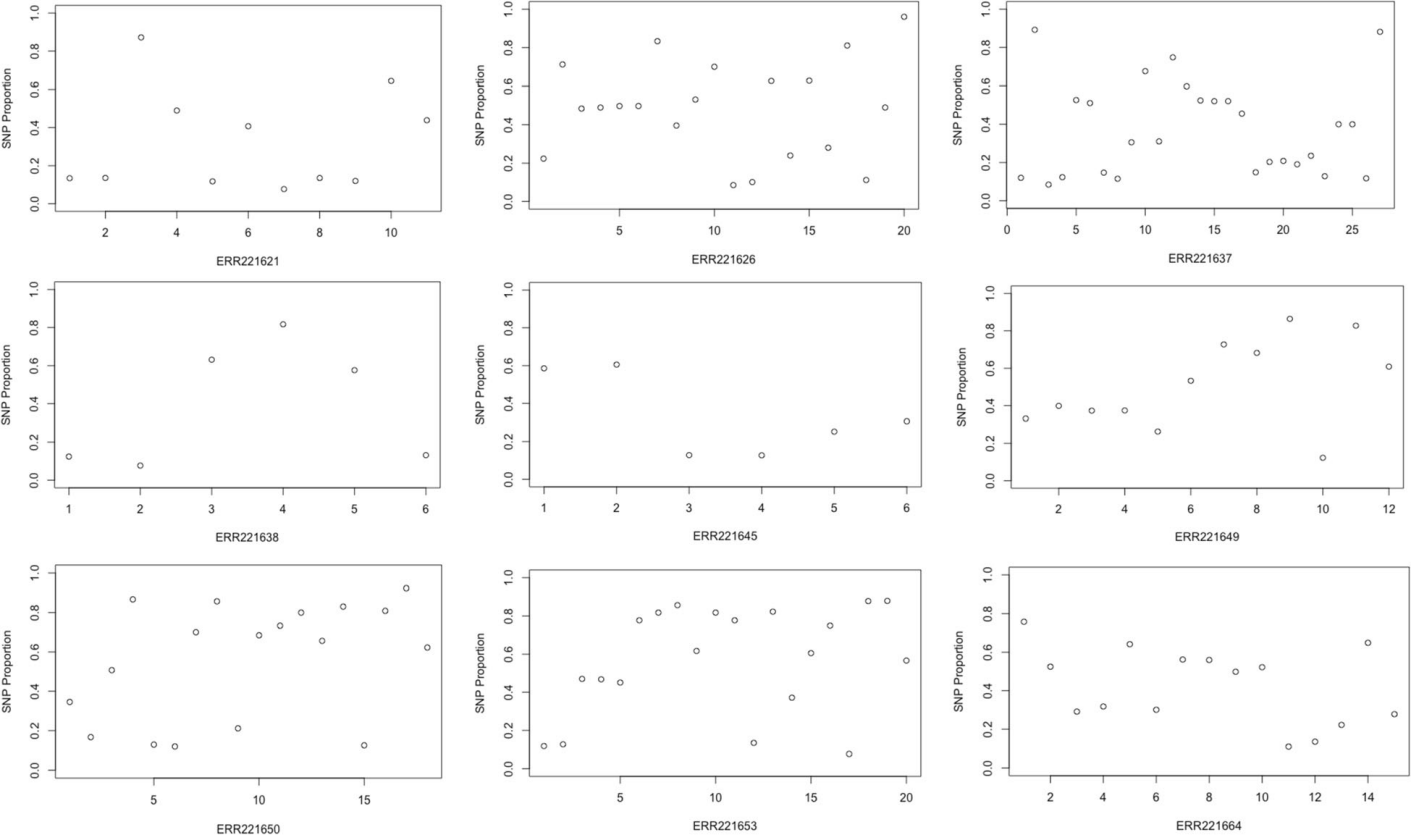
The plotted allele frequencies of reads at heterozygous sites in samples misidentified as pure strains in artificial mixtures of two strains using the Bayesian model-based clustering approach. The majority/minority strain proportions are 0.90 and 0.10 in sample ERR221649 and 0.95 and 0.05 in the remaining samples). The characteristic pattern of mixed infection that would be expected in samples of more than two non-clonal strains, e.g. Fig 1b, is not clear

The number of strains identified in each artificial mixture sample through Bayesian model-based clustering of heterozygous SNP read proportions is shown in Table 1. A BIC value of 20 was chosen as the maximum threshold for pure strains as this value identified all unmixed samples, and determined the highest number of mixed samples. All samples with a major proportion of 0.70 (12/12) and all but one with a major proportion of 0.90 (11/12) were correctly classified as containing two different strains, with all non-mixture samples identified as containing a single strain. The identification of mixtures in samples with 0.95 majority strain is again more difficult, with 8/12 samples misidentified as pure strains. In total, 9/36 mixed samples were misidentified as pure strains using this approach, performing worse than the heterozygous sites method (3/36 mixed samples misidentified). Closer inspection of these samples showed that there was no clear separation in allele frequencies at heterozygous sites, illustrated in Fig. 1, so they could not be delineated from pure strains. The allele frequencies at heterozygous sites in these samples are shown in Fig. 2.

**Fig. 3:**
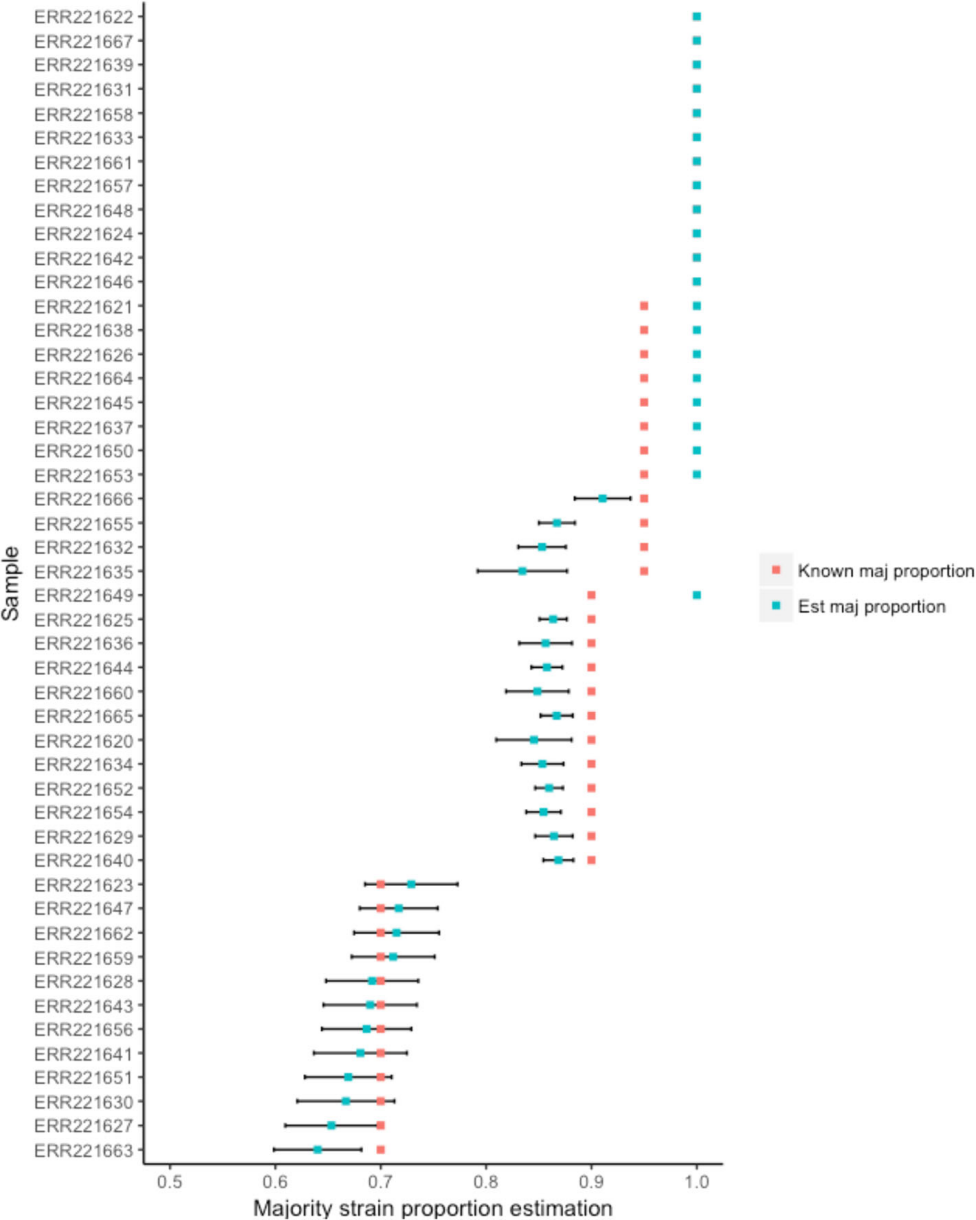
A comparison of the major strain proportion estimated through Bayesian model- based clustering (blue) against the known majority strain proportion (red) in all in vitro artificial mixture samples (*N* = 48). The standard deviation of allele frequencies of heterozygous sites around the mean of the estimated major proportion is shown by the error bars in black

The Bayesian mixture method also allows for an estimation of the mixing proportions of samples identified as mixed infection. All correctly classified mixed samples were found to contain two strains, with the mean of the uppermost cluster (closest to 1) a reasonable approximation of the majority strain proportion (Fig. 3). Differences in the estimated majority strain proportion to known mixture proportion ranged from 3.9–11.6% difference in mixtures with a 0.95 majority strain, 3.1–5.1% in mixtures with a 0.90 majority strain, and 0.08–6.0% in mixtures with a 0.70 majority strain.

### Identifying mixed infection in replicate samples

The robustness of the mixture detection methodologies employed in this work were inspected using replicate samples (Additional file 1). The dataset comprised one set of five biological replicates of the H37Rv reference strain and seven sets of three biological replicates of clinical Portuguese *M. tuberculosis* isolates. In addition, there were three sets of Portuguese TB isolates with six technical replicates and two further biological replicates.

Using the heterozygous sites method with a threshold of ≥20 sites, we identified four Portuguese samples as mixed infection, three biological replicates of the same sample (Por10, 14–19 heterozygous sites, heterozygous-total proportion between 1.6–2.2%) and one biological replicate of Por7 (14 heterozygous sites, heterozygous-total proportion 1.8%), with other Por7 replicates identified as pure strains. All replicate samples were identified as pure strains using the Bayesian clustering approach, including the four samples deemed mixed infection using the heterozygous sites method.

A table showing the sensitivity and specificity of both the heterozygous sites and Bayesian clustering approaches with the artificial mixture and replicate samples is shown in Table 2. At present, there is no gold standard test for detecting mixed infection in *M. tuberculosis* from WGS data. Therefore, true positives were taken as the artificially mixed Malawi samples that were known to be mixed infections (major strain proportion of 0.7, 0.9 and 0.95 in Table 1), and the true negative samples as the pure Malawi strains (major strain proportion 1.0 in Table 1), and all H37Rv and Portuguese *M. tuberculosis* replicate samples. The heterozygous sites method had a higher sensitivity than the Bayesian clustering method in detecting the true positive rate of mixed infections from the artificially mixed samples (91.7 to 75.0%); whereas the specificity of the Bayesian clustering method was the highest for identifying unmixed, pure samples (100% Bayesian to 93.5% heterozygous sites method).

**Table 2:** The sensitivity and specificity of the heterozygous sites and Bayesian model-based clustering approaches for detecting mixed infection in artificial mixture and replicate samples. Calculations assume that the 4 technical replicates of one sample that were classified as mixed by the heterozygous sites method came from a pure sample. True positives were taken as the known artificially mixed Malawi samples (Table 1), and true negatives as the known pure Malawi samples (Table 1), and all H37Rv and Portuguese replicate strains (Additional file 1)

	Number of mixed samples detected	
	Heterozygous sites method	Bayesian model-based clustering
Artificial mixed Malawi samples	33/36	27/36
Pure Malawi samples	0/12	0/12
Technical replicates	0/18	0/18
Biological replicates	4/32	0/32
**Sensitivity of method**	**91.7%**	**75.0%**
**Specificity of method**	**93.5%**	**100%**

### In silico artificial mixtures

**Fig. 4:**
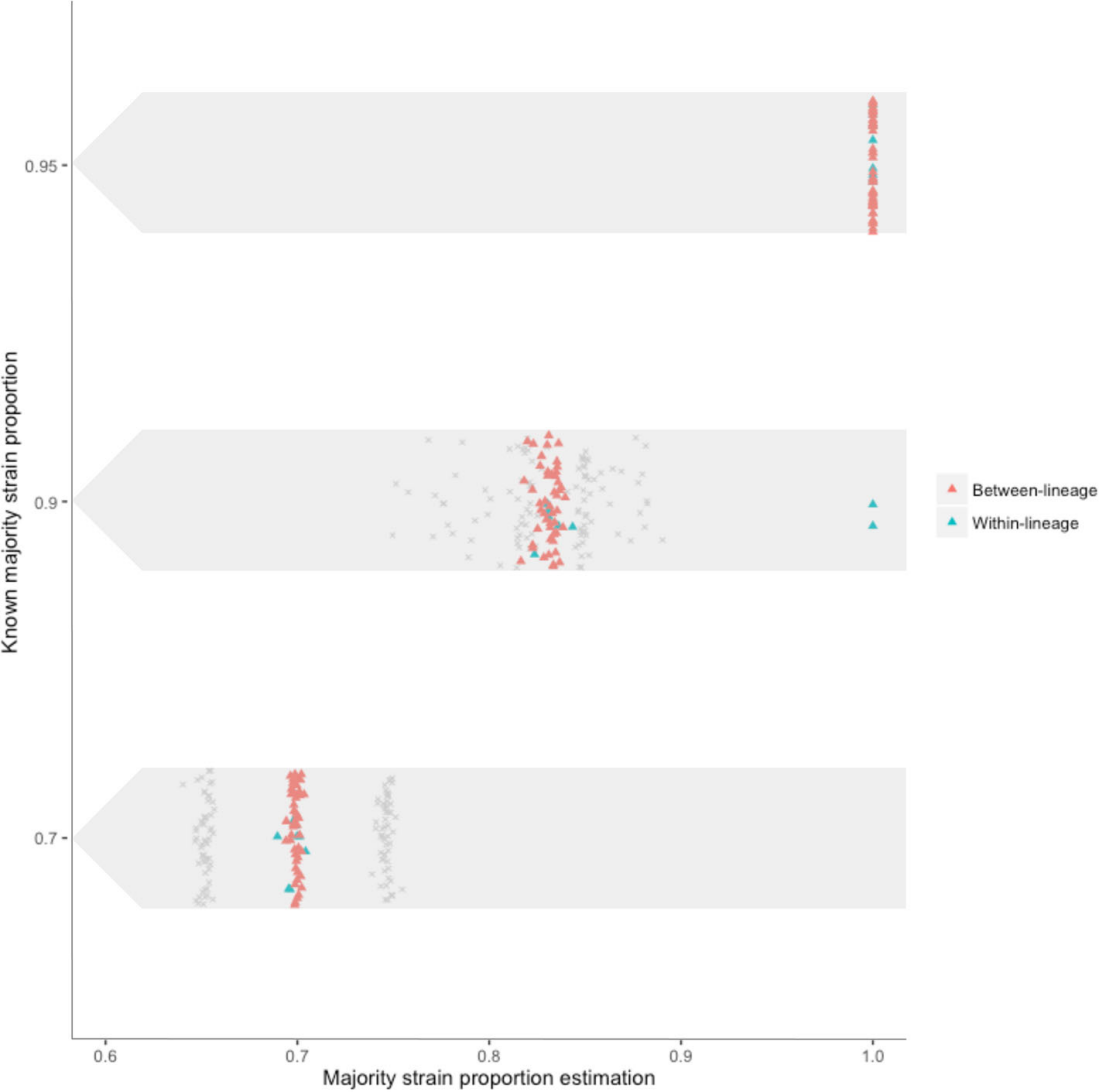
A comparison of the major strain proportion estimated through Bayesian model- based clustering against the known majority strain proportion in the in silico two-strain mixture samples (*N* = 168). The between-lineage samples are shown in red while the within-lineage samples are shown in blue. The standard deviation of allele frequencies of heterozygous sites around the mean of the estimated major proportion is shown by the grey crosses

A final evaluation of both the heterozygous sites and Bayesian clustering methods was carried out using to 168 in silico mixed samples (and the pure parental strains) with a priori known mixture proportions of 0.70/0.30, 0.90/0.10 and 0.95/0.05 (Additional file 1). All samples in the 0.70/30 proportion (56/56) and 96% of the 0.90/0.10 proportion (54/56) mixtures were correctly identified (Fig. 4). The mean majority strain proportion estimated using the Bayesian clustering method was 0.70 (SD 0.05) and 0.83 (SD 0.04) for the 0.70/0.30 and 0.90/0.10 mixtures respectively. The two 0.90 majority strain mixed samples that were not correctly identified were within-lineage mixes, one each of lineages 3 and 4, with only 8 and 2 heterozygous sites identified. None of the 0.95/0.05 mixed samples were identified as mixtures due to the low numbers of heterozygous sites found in these samples (between 0 and 2 sites in all samples) (Additional file 1).

### Malawi clinically-derived isolates

A clinical dataset comprising 1963 whole genome sequences from Malawi patients (one sample per infected host) covering lineages 1–4, as well as 5 *M. bovis* samples were then used to assess the prevalence of mixed infection in this population. Both the heterozygous sites and Bayesian clustering approaches were applied to this dataset to identify isolates likely to be mixed infection.

There was high concordance between the number of mixed infections identified with the heterozygous sites (195/1963; 9.9%) and Bayesian clustering methods (186/1963; 9.5%) (Additional file 1). With the heterozygous proportion approach, all clinical isolates with > 10 heterozygous sites also had a heterozygous proportion of > 1.5%, thus the number of heterozygous sites was the classifying factor with these samples using this approach.

**Fig. 5:**
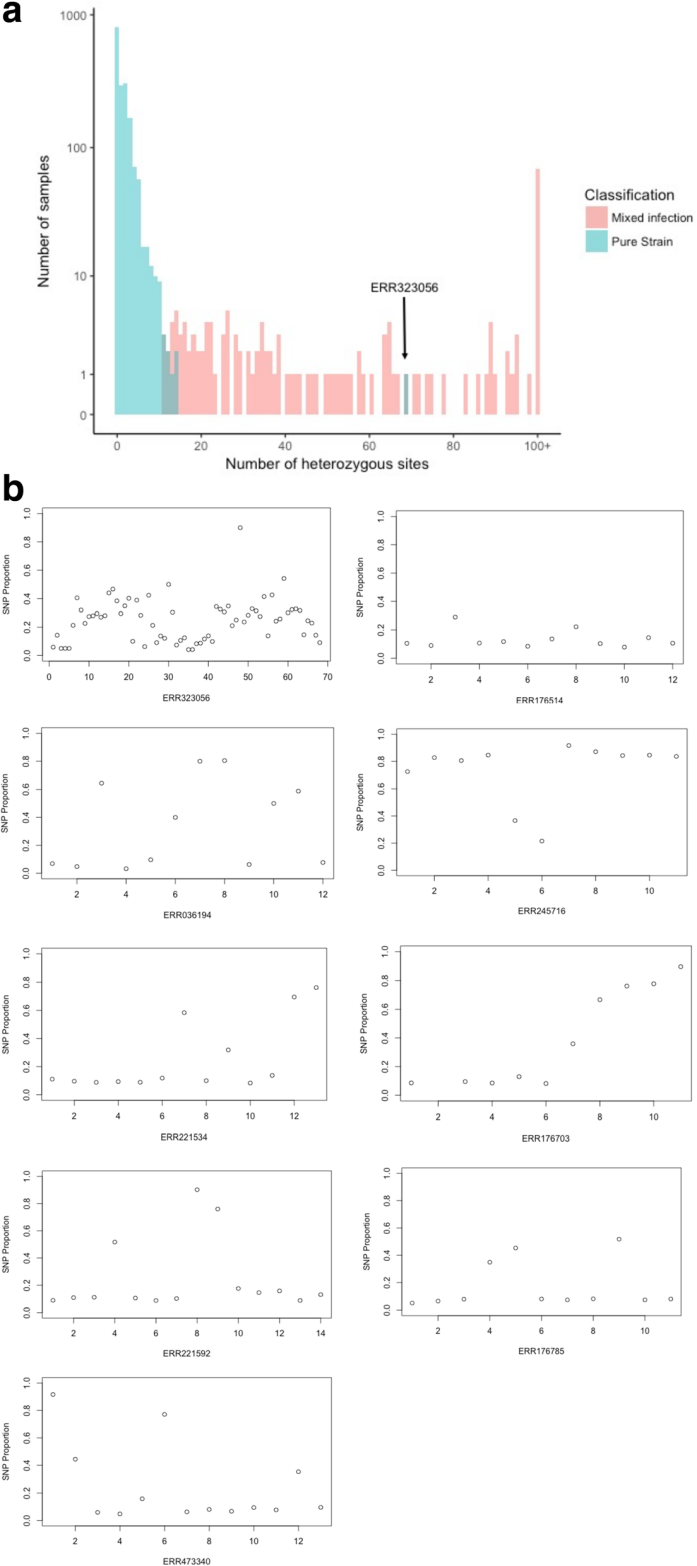
A closer inspection of samples identified as pure with the Bayesian clustering approach but mixed with the heterozygous sites approach. **a** A frequency histogram of heterozygous sites in Malawi samples identified as mixed infection or pure strains with the Bayesian clustering approach. Sample ERR323056, classified as a pure strain with 69 heterozygous sites, is highlighted. **b** The plotted allele frequencies of reads at heterozygous sites for samples identified as mixed using heterozygous sites approach but as pure strains with the Bayesian clustering approach, with sample ERR323056 shown first. Although there is some evidence of the characteristic pattern of mixed infection in some samples, the signal from heterozygous sites is insufficient to identify these strains as mixed infections

There were nine occurrences where mixed infections were found using the heterozygous sites approach, but samples were deemed single strains when applying the Bayesian clustering method; no samples were identified as mixed only by the Bayesian method. Of these nine isolates, eight had 11–14 heterozygous SNPs and heterozygous proportions of 1.7–3.3, and one had 69 SNPs and a heterozygous proportion of 12.38. Figure 5a shows a frequency histogram for the number of heterozygous sites found in all samples with the classification of mixed infection or pure strain through the Bayesian clustering method. Allele frequency of reads at heterozygous sites plots for the nine discrepant samples are shown in Fig. 5b.

### Associations with mixed infection

The association between mixed infections and demographic and disease features was investigated in the Malawi clinical isolates, including year of collection, age group of patient, sex of patient, HIV status, previous TB episode, lineage, type of TB (smear +/− and pulmonary), clinical outcome, and isoniazid and rifampicin resistance. Results are shown in Table 3.

**Table 3:** Tuberculosis disease characteristics associated with mixed infection. Nine individuals with mixed infections based on heterozygous sites but not with the Bayesian clustering method were excluded

Characteristic	Mixed / Total	% mixed	*P*-value
Year			
1995–1999	46/539	8.5	
2000–2004	82/662	12.4	
2005–2009	44/506	8.7	
2010–2014	14/247	5.7	0.009
Age group (years)			
< 15	2/34	5.9	
15–29	61/586	10.4	
30–44	87/866	10.1	
45+	36/468	7.7	0.4
Sex			
Female	94/995	9.5	
Male	92/959	9.6	0.9
HIV status			
Negative	52/636	8.2	
Positive	69/754	9.2	
Positive on ART	10/120	8.3	0.8
Unknown	55/444	12.4	
Previous TB			
No	173/1830	9.5	
Yes	13/124	10.5	0.7
Lineage			
1	35/314	11.2	
2	14/80	17.5	
3	15/212	7.1	
4	122/1343	9.1	
*M. bovis*	0/5	0.0	0.08
Type of TB			
Smear +	125/1462	8.6	
Smear -	53/400	13.3	
Extra-pulmonary	8/92	8.7	0.02
Outcome			
Completed	116/1275	9.1	
Died	41/405	10.1	
Lost/transferred	29/274	10.6	0.7
Isoniazid resistance			
Resistant	15/130	11.5	
Sensitive	169/1757	9.3	0.5
Rifampicin resistance			
Resistant	2/17	11.8	
Sensitive	182/1870	9.7	0.7

*P* values from chi-square test except for associations with rifampicin resistance for which the Fisher exact test was used because of small numbers; *ART* Antiretroviral therapy

Of the possible risk factors assessed, only the year of collection has a significant association with mixed infection of TB strains (*p* = 0.009). Patients with smear-negative pulmonary tuberculosis (SNPT) were also found to be more likely to harbour a mixed infection than patients smear-positive pulmonary tuberculosis.

(SPPT) and extra-pulmonary tuberculosis (*p* = 0.02). No other disease characteristics were found to be significantly associated with mixed infection.

## Discussion

We have developed methods that can be used to detect the signals of mixed infection in *M. tuberculosis* from whole genome sequence data. These methods can be performed in silico without requiring laboratory testing, which can often be labour intensive and costly, allowing for a rapid exploration of large datasets. We found that the signal from heterozygous sites alone was sufficient to identify mixtures in both artificially mixed and clinically-derived samples, with mixed infection confidently predicted in samples with a low number of heterozygous sites (12 and 11 SNPs with the heterozygous sites and Bayesian clustering approaches). Therefore, considering variation within whole genome sequence data allows mixed infections of closely related strains, such as those from within the same lineage or genotype, to be identified.

There were key differences between the heterozygous sites and Bayesian clustering approaches that led to different numbers of mixed samples being reported in different datasets. In the artificial *in vitro* mixed samples, we found that the heterozygous sites method had better sensitivity in detecting mixed samples, with only 3/36 mixtures not identified compared to 9/36 samples misidentified using Bayesian clustering. The signal from the allele frequencies of reads in these samples was indistinguishable from clonal heterogeneity that could be found in pure samples and so the Bayesian clustering could not effectively identify the characteristic patterns of mixed infection in these samples.

In the replicate samples, the heterozygous sites method identified four samples as mixed infection that were not found to be mixed using the Bayesian clustering method. All replicate samples were considered pure strains before analysis, though all three biological replicates of one Portuguese isolate were identified as mixed infection with the heterozygous sites approach. The Bayesian clustering approach did not support this classification. In these cases, as well as with the nine samples in the clinical Malawi dataset where there was a different classification between detection methods, it may be that an isolate has relatively high levels of clonal variability, resulting in false-positives when using the heterozygous sites approach.

The Portuguese samples were either multidrug or extensively-drug resistant and, while SNPs in known drug resistance loci were removed from the analysis, other associated sites that were under selection may have been retained that appear as heterozygous sites. Allele frequencies at sites under selection can be highly variable over time and through treatment in TB infections [29]. Consequently, drug resistant samples may have a relatively high number of heterozygous sites with variable allele frequencies. These samples will be correctly differentiated from mixed infections where allele frequencies at heterozygous sites will be consistent across the genome by the Bayesian clustering method, but may be incorrectly identified as mixed infections with the heterozygous sites method. Multidrug resistance has also been linked to increased mutation rates and hyper-mutant strains in TB, particularly in ‘Beijing’ strains [30, 31], which may also increase levels of heterogeneity in clonal isolates and lead to samples incorrectly classified as mixed infection when using the number of heterozygous sites alone. As such, it appears that the heterozygous sites method is more sensitive in identifying mixed infection but may overestimate the number of mixed infections in a population. The Bayesian clustering method though will have a lower sensitivity in detecting mixed infection but a higher specificity in correctly identifying pure strains.

Samples where the minority strain proportion was very low proved more difficult to accurately identify in both the *in vitro* and in silico artificially mixed samples, and this problem has been highlighted in previous attempts to detect mixed infection [4, 5]. In the *in vitro* artificial mixtures with a majority strain proportion of 0.95, only 9/12 could be identified as mixed infection with heterozygous proportions, and 4/12 identified through Bayesian clustering. The samples correctly identified as mixtures in 0.95/0.05 ratios were either between lineage mixes or mixtures between two strains of the highly diverse Beijing genotype.

No in silico artificial mixtures with a 0.05 minority proportion were able to be identified compared to pure strains as the number of heterozygous sites in these samples was found to be very low (between 0 and 2 sites across all 56 samples). Inspecting the raw alignment files at sites that differed between the two parental strains, and thus would be heterozygous sites, it appears as though the signal from the minority strain was indistinguishable from sequencing error and so were instead called as the allele given by the majority strain. We chose to set the sequencing error in these simulated genomes as relatively high, reflecting the top estimates of Illumina HiSeq error rates, though manual inspection of our clinically-derived KPS samples and *in vitro* mixed samples showed a lower frequency of sequence errors. As sequencing technologies continue to improve and the error rate decreases, we predict that mixed samples with lower minority allele frequencies will be able to be identified.

Analysing 1963 clinical *M. tuberculosis* isolates from the Karonga Prevention Study in Malawi with both the heterozygous sites and Bayesian clustering methods we found evidence of mixed infection in between 9.5–9.9% of the population. We had previously identified a proportion of mixed infections of 2.8% in this population looking only at mixtures between LAM and Beijing strains [6]. The incidence of mixed infection found in Malawi is lower than has been identified in samples from Cape Town, South Africa (19% between Beijing and non-Beijing strains) [32], consistent with the much higher incidence of tuberculosis in South Africa [18, 33], with TB incidence suggested to be linked to the rate of mixed infection [6, 7].

Additionally, the rate of mixed infection in South Africa was estimated using RFLP and spoligotype analysis directly from sputum, whereas our methods have used whole genome data isolated from solid culture. Isolating DNA directly from sputum will likely provide a more representative sample of the full range of strains present as culturing can result in differential selection of strains. At present, the application of sequencing directly from sputum samples has been mainly limited to the rapid identification of *M. tuberculosis* from diagnostic markers, though recent work has obtained high quality whole genome sequence data at a suitable depth of coverage for the application of our methods for detecting mixed infection [34].

Interestingly there were few associations identified with the presence of mixed TB infection. The peak proportion between 2000 and 2004 is consistent with the peak incidence of TB in the district a few years earlier [35]. Until 2010, all isolates underwent several rounds of culture and subculture before DNA extraction. There is no evidence of a higher proportion of mixed infections in the post 2010 period when DNA extraction was performed from the first set of cultures. An association with smear negative TB could be a chance finding given the multiple comparisons.

Reconstructing the transmission of all samples and tracing contact networks would assist us to gain a better understanding of how mixed infections are acquired. The methods detailed here for identifying mixed infections can be extended to estimate an approximation of the parental strain genomes in mixtures by imputing the nucleotide base call that has come from major and minor strains in a mixed infection at each heterozygous site. Including these sequences in transmission reconstruction could provide a more complete picture of the spread of a pathogen by including transmission events from minor frequency strains.

It may prove more challenging to confidently detect mixed infection in organisms other than *M. tuberculosis* using the methods detailed in this paper, particularly in taxa with a high rate of recombination. *Mycobacterium* species are known to have very little recombination (excluding *pe/ppe* genes [36]) and strong clonal population structure [37]. One solution is to use the levels of heterozygosity at the gene-level or in larger genomic regions to look for the signatures of mixed infection. We found that these characteristic patterns of mixed infection are present in certain *Mycobacterium* Regions of Difference (RDs) in some mixed samples (Additional files 2 and 3), and so the methodologies described here could be applied to similar diagnostic marker regions in other taxa to estimate the presence of mixed infection.

These methods can be applied to identify mixed infection and characterise strain diversity across all sites within a host where DNA can be isolated, not limited to cultured sputum samples. This is particularly important with the evidence of the reduction in strain diversity from samples taken from the upper airway of patients as compared to in the lung, and strain heterogeneity across different sites within the lung itself [15]. Although we have found the rate of mixed infection in our clinical dataset of Malawian isolates to be relatively high (around 10%), this is still likely to be lower than the true rate of mixed infection as only sputum samples were taken, and many were subcultured. It is also possible that where samples are sequenced at a higher coverage the signal from minor strains present in a sample will be more evident, further increasing the number of mixed infections identified.

## Conclusion

In conclusion, we have presented simple methods for identifying mixed *M. tuberculosis* infections using variation in whole genome sequencing data. These analyses can help to accurately reconstruct the evolution and transmission of *M. tuberculosis* infections, or can be applied to individual cases where low frequency variants may be considered in the treatment of the disease.

## Additional files

Additional file 1: Strain information and full results table for clinically-derived Malawi samples, in silico replicate samples and Portuguese replicate samples. Lineage, total number of SNPs, number of heterozygous sites and the mixture analysis result for both Bayesian clustering and heterozygous sites approaches is included for each sample. (XLSX 162 kb)

Additional file 2: Analysis and interpretation of Regions of Difference (RD) analysis in clinically-derived Malawi strains. (DOCX 127 kb)

Additional file 3: Coverage plots illustrating four Regions of Differences (RDs) in clinically-derived Malawi strains. (PDF 404 kb)

## Abbreviations

BIC: Bayesian information criterion
HIV: Human Immunodeficiency virus
PCR: Polymerase chain reaction
RD: Region of difference
SNP: Single nucleotide polymorphism
TB: Tuberculosis
WGS: Whole genome sequencing
