# Supplementary material for: Identifying mixed Mycobacterium tuberculosis infections from whole genome sequence data
Source: BMC Genomics. 2018 Aug 14;19:613. doi: 10.1186/s12864-018-4988-z (PMC6092779; doi:10.1186/s12864-018-4988-z)
Supplement: Supplementary file 2 — Analysis and interpretation of Regions of Difference (RD) analysis in clinically-derived Malawi strains. (DOCX 127 kb) [file 12864_2018_4988_MOESM2_ESM.docx]

**Supplementary Results**

*Regions of difference*

In addition to using heterozygous SNPs to detect mixed infections, we also examined the read depth of coverage across selected Regions of Difference (RDs) in the samples to determine whether signals of mixed samples are also present in larger genomic regions, and whether these can be used to calculate the mixture proportions within artificial mixture samples. RDs are considered useful diagnostic markers to differentiate between *Mycobacterium* species and *M. tuberculosis* lineages (1, 2). Eighteen RDs were considered that have been found to be robust markers for distinguishing between *M. tuberculosis* lineages (3). For example, RD174, RD239, RD750, and RD105 deletions are used to specifically define isolates belonging to the “LAM”, “Indo-Oceanic,” “East African/Indian,” and “East Asian” (or “W/Beijing”) strain lineages, respectively.

Mapping the read depth of coverage in the artificial mixtures across selected RDs showed some instances where the signals of mixed infections could be detected in larger portions of the genome. As an example, in sample ERR221643 there were two RDs where the average read depth across two RDs (RD174 and RD750) dropped to levels comparable to the mixture proportion (70.97% and 29.96% respectively) when compared to the average coverage across the genome (**Figure S1a and S1b**). These patterns were also found in four additional RDs in up to 11 mixture samples of varying proportions.

These patterns were not seen across all mixed samples, with some showing no mixtures variation in coverage across any of the selected RDs. For instance, there was little variation in read depth of coverage across any RD in sample ERR221627 (majority strain proportion 0.70) (**Figure S1c)**, despite a relatively high proportion of heterozygous SNPs (43.43%) (**Table 1 – main text**), as this was a mixture of two strains within the same lineage (lineage 1) and RDs appear only to distinguish between strains within different lineages.

Examining the average read depth across some RDs can result in erroneous positive results. Taking the average coverage at RD207 in sample ERR221628, there appears to be some correlation between the read depth in this region and the proportion of the minor strain in the mix (23.98% and 0.30). Mapping this coverage though shows that the read depth across the RD is highly variable, and similar patterns can be observed in samples with a lower mixture proportion or single strain (**Figure S1d).**

Additionally, the read depth of coverage was highly variable between samples at the same RD, thus making it difficult to discern true mixed samples, particularly when minor strain proportions are very low, from standing coverage variation across the genome. Therefore, mapping read depth of coverage across RDs can help to validate some instances where samples may be considered mixtures, though application appears likely to be limited to mixtures between lineages and lacks sensitivity in detecting mixtures where strains are in highly different proportions.

**Supplementary references**

1. Brosch R, Gordon S V., Marmiesse M, Brodin P, Buchrieser C, Eiglmeier K, et al. A new evolutionary scenario for the Mycobacterium tuberculosis complex. Proceedings of the National Academy of Sciences. 2002;99:3684–9. doi:10.1073/pnas.052548299.

2. Tsolaki AG, Hirsh AE, DeRiemer K, Enciso JA, Wong MZ, Hannan M, et al. Functional and evolutionary genomics of Mycobacterium tuberculosis: insights from genomic deletions in 100 strains. Proceedings of the National Academy of Sciences of the United States of America. 2004;101:4865–70.

3. Faksri K, Xia E, Tan JH, Teo Y-Y, Ong RT-H. In silico region of difference (RD) analysis of Mycobacterium tuberculosis complex from sequence reads using RD-Analyzer. BMC Genomics. 2016;17:847. doi:10.1186/s12864-016-3213-1.
