# Supplementary material for: Identifying mixed Mycobacterium tuberculosis infections from whole genome sequence data
Source: BMC Genomics. 2018 Aug 14;19:613. doi: 10.1186/s12864-018-4988-z (PMC6092779; doi:10.1186/s12864-018-4988-z)
Supplement: Supplementary file 3 — Coverage plots illustrating four Regions of Differences (RDs) in clinically-derived Malawi strains. (PDF 404 kb) [file 12864_2018_4988_MOESM3_ESM.pdf]

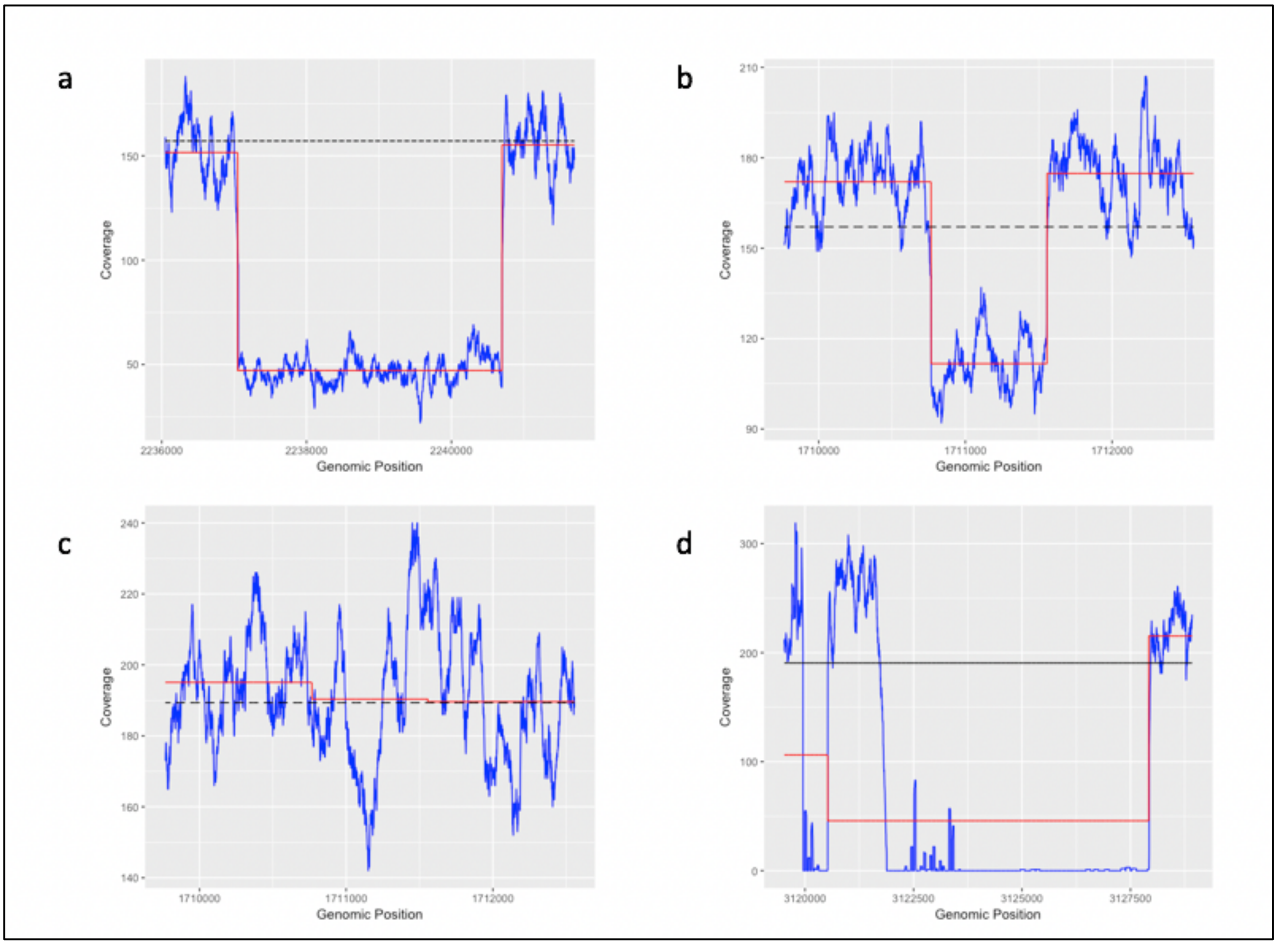

Figure S1. Plots showing the read depth of coverage over selected Regions of Difference (RDs). Regions 1000bp upstream and downstream of the RD are also included. All samples shown are artificial mixtures of two strains. a) RD174, sample identifier ERR221643 (0.70 major strain); b) RD750, sample identifier ERR221643 (0.70 major strain); c) RD750, sample identifier ERR221627 (0.70 major strain); and d) RD207, sample identifier ERR221631 (1.0 major strain). Blue lines indicate the base specific coverage, red lines are the average coverage across the RD and 1000bp upstream and downstream, and the black dashed lines indicate the average coverage across the whole genome.
